# Swept-source Raman spectroscopy of chemical and biological materials

**DOI:** 10.1117/1.JBO.29.S2.S22703

**Published:** 2024-04-03

**Authors:** Jeonggeun Song, Peter T. C. So, Hongki Yoo, Jeon Woong Kang

**Affiliations:** a Korea Advanced Institute of Science and Technology, Department of Mechanical Engineering, Daejeon, Republic of Korea; b Laser Biomedical Research Center, G. R. Harrison Spectroscopy Laboratory, Massachusetts Institute of Technology, Cambridge, Massachusetts, United States

**Keywords:** swept-source laser, signal processing, Raman spectroscopy, narrow bandwidth filter, acetaminophen, glucose, phenylalanine, hydroxyapatite, swine tissue, biological tissue mapping, low-noise silicon photoreceiver

## Abstract

**Significance:**

Raman spectroscopy has been used as a powerful tool for chemical analysis, enabling the noninvasive acquisition of molecular fingerprints from various samples. Raman spectroscopy has proven to be valuable in numerous fields, including pharmaceutical, materials science, and biomedicine. Active research and development efforts are currently underway to bring this analytical instrument into the field, enabling *in situ* Raman measurements for a wider range of applications. Dispersive Raman spectroscopy using a fixed, narrowband source is a common method for acquiring Raman spectra. However, dispersive Raman spectroscopy requires a bulky spectrometer, which limits its field applicability. Therefore, there has been a tremendous need to develop a portable and sensitive Raman system.

**Aim:**

We developed a compact swept-source Raman (SS-Raman) spectroscopy system and proposed a signal processing method to mitigate hardware limitations. We demonstrated the capabilities of the SS-Raman spectroscopy by acquiring Raman spectra from both chemical and biological samples. These spectra were then compared with Raman spectra obtained using a conventional dispersive Raman spectroscopy system.

**Approach:**

The SS-Raman spectroscopy system used a wavelength-swept source laser (822 to 842 nm), a bandpass filter with a bandwidth of 1.5 nm, and a low-noise silicon photoreceiver. Raman spectra were acquired from various chemical samples, including phenylalanine, hydroxyapatite, glucose, and acetaminophen. A comparative analysis with the conventional dispersive Raman spectroscopy was conducted by calculating the correlation coefficients between the spectra from the SS-Raman spectroscopy and those from the conventional system. Furthermore, Raman mapping was obtained from cross-sections of swine tissue, demonstrating the applicability of the SS-Raman spectroscopy in biological samples.

**Results:**

We developed a compact SS-Raman system and validated its performance by acquiring Raman spectra from both chemical and biological materials. Our straightforward signal processing method enhanced the quality of the Raman spectra without incurring high costs. Raman spectra in the range of 900 to $1200\;{\mathrm{cm}}^{-1}$ were observed for phenylalanine, hydroxyapatite, glucose, and acetaminophen. The results were validated with correlation coefficients of 0.88, 0.84, 0.87, and 0.73, respectively, compared with those obtained from dispersive Raman spectroscopy. Furthermore, we performed scans across the cross-section of swine tissue to generate a biological tissue mapping plot, providing information about the composition of swine tissue.

**Conclusions:**

We demonstrate the capabilities of the proposed compact SS-Raman spectroscopy system by obtaining Raman spectra of chemical and biological materials, utilizing straightforward signal processing. We anticipate that the SS-Raman spectroscopy will be utilized in various fields, including biomedical and chemical applications.

## 1 Introduction

Raman spectroscopy is a powerful analytical tool, providing molecular fingerprints of samples by measuring the changes in frequency and the intensity of inelastically scattered light.^1^^,^^2^ The frequency shifts resulting from energy changes are indicative of molecular bonding and vibrational modes. Inelastically scattered light is categorized into two types: (1) anti-Stokes scattering with energy higher than the incident light and (2) Stokes scattering with lower energy.^3^ Because Stokes scattering is generally much stronger than anti-Stokes scattering, most Raman spectroscopy focuses on Stokes scattering, even though anti-Stokes scattering is almost free from background interference.

Raman spectroscopy has proven its efficacy in various fields, including pharmaceutical^4^^,^^5^ and materials science.^6^ Notably, due to its capability for noninvasive, *in situ* chemical analysis, Raman spectroscopy is widely used in biological and biomedical applications.^7^ It analyzes substances such as acetaminophen,^8^ glucose,^9^ phenylalanine,^10^ and hydroxyapatite^11^ and has been used for clinical diagnostic applications in diabetes,^12^^,^^13^ coronary artery disease,^14^ and cancer.^15^

Dispersive Raman spectroscopy is a commonly used method for measuring Raman spectra. It utilizes a fixed-wavelength light source and a spectrometer. Dispersive Raman spectroscopy enables the simultaneous acquisition of the entire Raman spectrum using a line charge coupled device (CCD) or rectangular CCD. Because a small amount of Raman photons is dispersed over a large spectral region, Raman spectra can be obtained by detecting Raman photons with a high throughput spectrometer. However, high throughput Raman spectroscopy systems tend to be bulky due to the larger optical components and detector.^16^ The substantial size of the spectroscopy system poses a challenge in developing portable Raman spectroscopy. Commercial portable Raman spectroscopy systems, such as the mini-spectrometer (C14214MA, Hamamatsu, Japan) and handheld Raman spectrometer (BRAVO, Bruker, Billerica, Massachusetts, United States) have been developed; however, they are more geared to chemical analysis and not sensitive enough for many biomedical applications. Many studies have concentrated on strategies such as increasing the excitation power, using longer acquisition times, and employing cooled detectors to enhance Raman spectra.^17^ Nevertheless, these approaches may result in sample damage and incur high costs. Furthermore, in a recent study, the successful implementation of compact Raman spectroscopy with a picosecond laser, time-correlated photon counting detection, and a single-mode fiber significantly decreased the weight in comparison to conventional Raman spectroscopy employing a CCD-based detector.^18^

The wavelength-tunable swept-source laser has emerged as a critical component in biomedical imaging. Swept-source optical coherence tomography (SS-OCT) has been applied in various biomedical applications, such as intravascular imaging.^19^^,^^20^ Additionally, two-photon microscopy has also been demonstrated using a swept-source laser.^21^ In this study, we developed a Raman spectroscopy system utilizing a swept-source laser, departing from the conventional fixed-wavelength light source. Swept-source Raman (SS-Raman) spectroscopy was initially proposed in a patent (US9662047B2, portable Raman diagnostic system) as a way to implement a compact Raman system. This patent also describes the integration of OCT and Raman using a single swept-source. Recently, Atabaki et al.^22^ demonstrated SS-Raman spectroscopy by combining a MEMS-tunable vertical cavity surface emitting laser with a bandpass filter having a 0.5 nm bandwidth. However, further improvements, such as increasing the optical power and sample mapping capabilities, are still required to overcome limitations and enable wider applications, especially in biomedicine.

In this study, we developed a compact SS-Raman spectroscopy system utilizing a swept-source laser, a bandpass filter, and a highly sensitive silicon photoreceiver. The integration of these components enables the implementation of a cost-effective and compact Raman spectroscopy system. Moreover, we introduced a signal processing technique to enhance the Raman spectra by eliminating artifacts and minimizing signal-broadening effects, which can be achieved without incurring high costs. To validate the SS-Raman spectroscopy, we obtained Raman spectra from diverse chemical samples, including phenylalanine, hydroxyapatite, glucose, and acetaminophen, and compared them with spectra from conventional dispersive Raman spectroscopy. Additionally, we acquired Raman spectra from swine tissue cross-sections to generate biological tissue mapping plots, demonstrating the applicability of the SS-Raman spectroscopy in biological samples.

## 2 Methods and Materials

### 2.1 Principle of SS-Raman Spectroscopy

The recently proposed SS-Raman uses a swept-source laser, a narrow-bandwidth bandpass filter, and a highly sensitive point photoreceiver for acquiring Raman spectra.^22^ These components contribute to the development of compact and cost-effective Raman spectroscopy systems. The narrow linewidth of the swept-source laser sequentially tunes the wavelength across the light source’s wavelength range. SS-Raman spectroscopy takes advantage of the independence of Raman shift from the excitation wavelength,^23^ resulting in a consistent nonresonant Raman spectral shape obtained during the wavelength sweep. Because the wavelength of the Raman signal changes during the sweep, the use of a narrow-bandwidth filter and a highly sensitive photoreceiver enables the acquisition of the desired Raman spectra, which are determined by the difference between the center wavelength of the bandpass filter and the excitation wavelength. The Raman shift in SS-Raman spectroscopy is expressed as follows:

$$\text{Raman shift }({\mathrm{cm}}^{-1})=\frac{1}{\lambda_{\mathrm{ext}}}-\frac{1}{\lambda_{\mathrm{bp}}},$$ (1)

where $\lambda_{\mathrm{ext}}$ is the excitation wavelength, and $\lambda_{\mathrm{bp}}$ is the center wavelength of the bandpass filter. Compact SS-Raman spectroscopy operates on time-sequential detection to obtain Raman spectra. While it cannot obtain the entire spectrum simultaneously as dispersive Raman spectroscopy can, the high sensitivity of the point detector effectively mitigates this limitation.

### 2.2 SS-Raman Spectroscopy System and Components

**Fig. 1:**
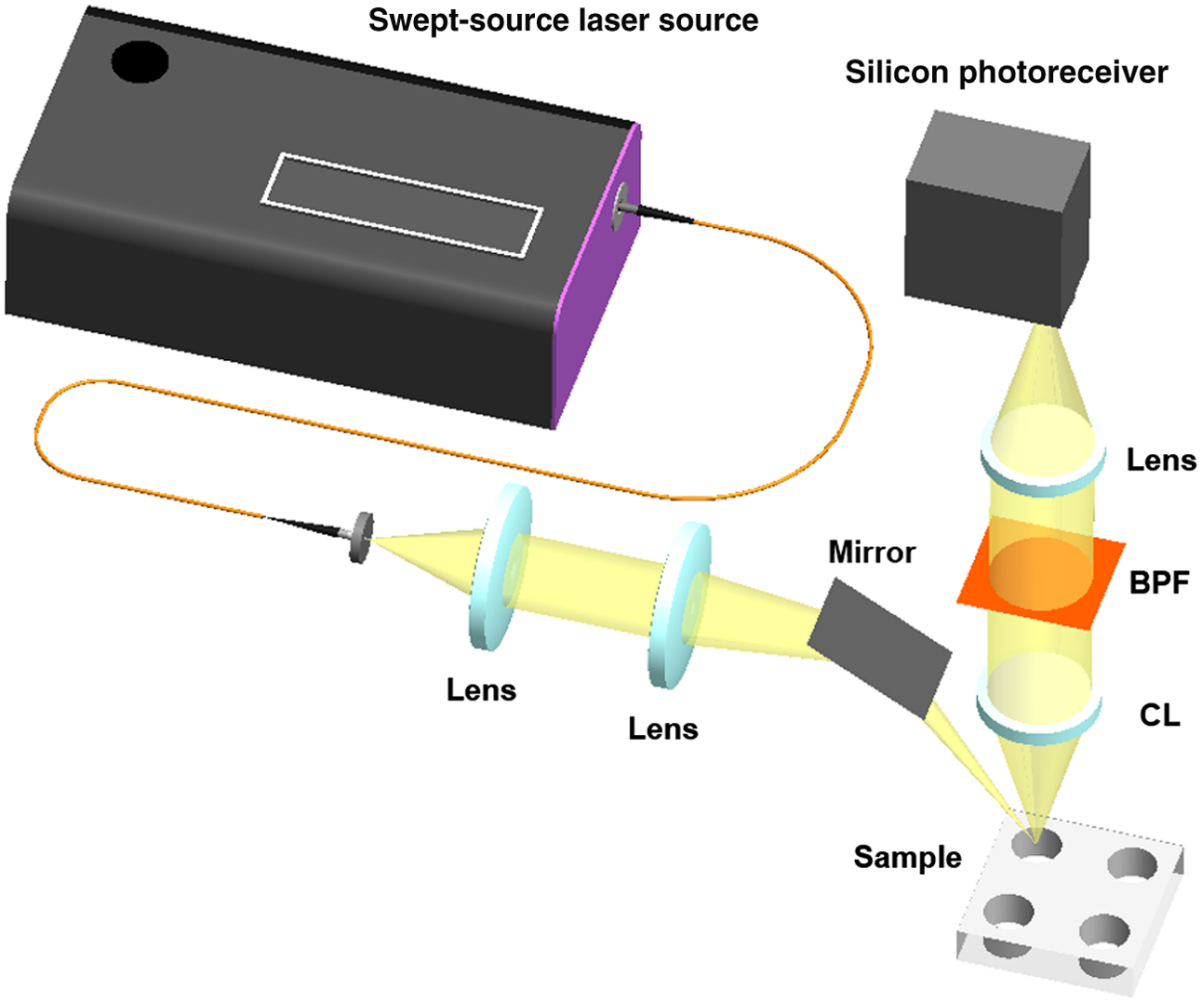
Schematic diagram of the SS-Raman spectroscopy system. A wavelength-swept laser, ranging from 822 to 842 nm, coupled with a multimode optical fiber, was utilized. The sample was illuminated with converging lenses and a mirror. Raman signals were collected through a collection lens and detected by a photoreceiver. The Raman spectra were acquired during a single sweep using a narrow-bandwidth bandpass filter. BPF, bandpass filter; CL, collection lens.

Figure 1 illustrates the compact SS-Raman spectroscopy system developed in this study. The schematic provides an overview of the entire SS-Raman spectroscopy system, which comprises two main parts: one for sample excitation and another for collecting the scattered Raman signals from the samples. The system utilizes a swept-source laser (XPERAY, Nanobase, Seoul, Republic of Korea) that sweeps the wavelength range from 822 to 842 nm with an instantaneous linewidth of 10 pm. The output laser power, sweeping wavelength interval, and sweeping speed are controlled by the laser software. For Raman spectra acquisition, we used an excitation power of 100 mW and a wavelength sweep interval of 0.05 nm with each interval having an exposure time of 0.1 s, resulting in a total acquisition time of 40 s for 400 wavelength steps. The laser output, delivered through a multimode fiber, is collimated by a lens (AC254-100-B, Thorlabs, Newton, New Jersey, United States), filtered through an 850 nm short-pass filter (FESH0850, Thorlabs, United States) to eliminate background noise and scattered light from the fiber and then focused onto the sample using a focusing lens (AC254-150-B, Thorlabs, United States). A silver mirror directs the excitation beam to the sample stage. The Raman scattering signals from the sample were collected by a collection lens (AC254-050-B, Thorlabs, United States) with a numerical aperture (NA) of 0.25. An 850 nm long-pass filter (FELH0850, Thorlabs, United States) is utilized to obtain Raman signals without Rayleigh scattering. Additionally, a 912 nm bandpass filter with a bandwidth of 1.5 nm (912-1.5 OD4 Ultra Narrow Bandpass filter, Alluxa, Santa Rosa, California, United States), located after the long-pass filter, facilitates the acquisition of Raman spectra during a single sweep of the light source. According to Eq. (1), the range of the acquired Raman spectra spans from 900 to $1200\;{\mathrm{cm}}^{-1}$. A converging lens (AC254-050-B, Thorlabs, United States) focuses the Raman signal onto the active area of the ultra-low noise and high-sensitive silicon detector (FWPR-20-Si, FEMTO, Berlin, Germany). Data are acquired via a data acquisition (DAQ) board and a PC, and the Raman spectra are monitored in real-time through a LabVIEW program.

## 3 Results

### 3.1 Hardware Limitation and Signal Processing

The developed SS-Raman spectroscopy system faced practical hardware limitations that adversely affected the quality of the Raman spectra. Notably, the use of a bandpass filter with a relatively broad bandwidth in our system led to compromised spectral resolution and broadened Raman peaks. Additionally, the unstable output power of the swept-source laser introduced ripple noises into the Raman signal, and the low optical density of the Rayleigh rejection filters contributed to a significant background signal. Although the approach of using high-performance light sources and ultra-narrow bandpass filters could mitigate these hardware limitations, it comes with a substantial cost. In response, our study proposed a straightforward signal processing method aimed at enhancing the Raman signal without incurring significant expenses.

**Fig. 2:**
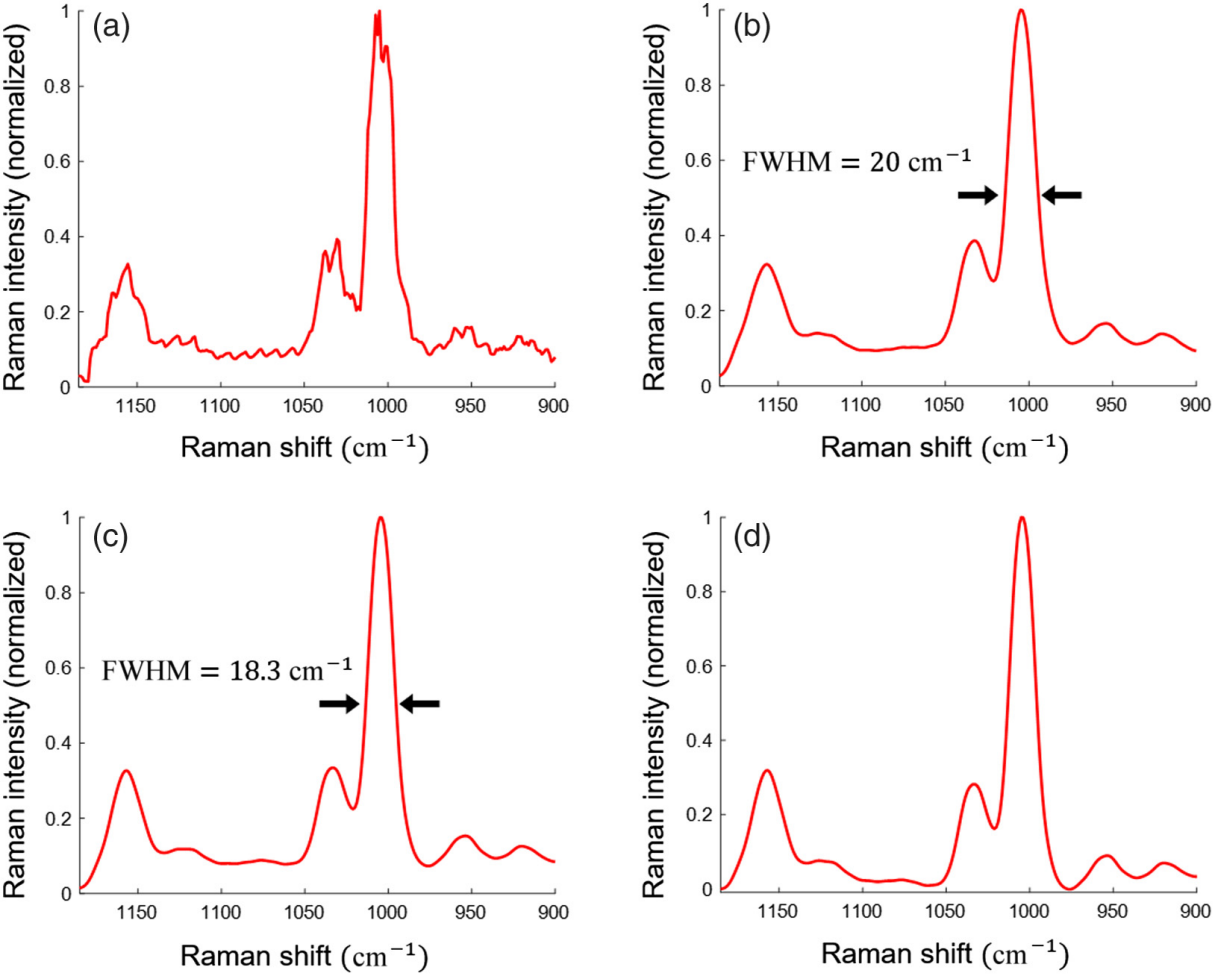
Signal processing in SS-Raman spectroscopy. (a) Raw Raman spectrum of phenylalanine powder shows degradation due to the unstable output of the light source and the use of filters with a large bandwidth. (b) Application of Gaussian filtering effectively eliminates high-frequency noise. (c) Deconvolution enhances the spectral resolution. (d) Polynomial background removal mitigates the background signal arising from the low optical density of the filter.

Figure 2 shows the signal processing process to improve the Raman spectra. Figure 2(a) shows the raw Raman signal of phenylalanine powder measured by SS-Raman spectroscopy. A sawtooth-like ripple noise is introduced due to the instability of the light source. Gaussian filtering was applied, to eliminate this high-frequency noise, including the ripple noise while preserving the Raman signal, as shown in Fig. 2(b). However, both the Gaussian filtering and the broad bandwidth of the bandpass filter contribute to the broadening of the Raman peaks. The full width at half maximum (FWHM) of the $1004\;{\mathrm{cm}}^{-1}$ Raman peak of phenylalanine was $20\;{\mathrm{cm}}^{-1}$. The deconvolution method can be used to improve the spectral resolution of the instrumentally broadened Raman spectra caused by the slit in dispersive Raman spectroscopy.^24^ Figure 2(c) shows the enhanced spectral resolution after deconvolution, considering both the bandpass filter transmission and the Gaussian filter, reducing the FWHM of the $1004\;{\mathrm{cm}}^{-1}$ Raman peak of phenylalanine to $18.3\;{\mathrm{cm}}^{-1}$—an improvement of the spectral resolution by 8.5%. Finally, Fig. 2(d) shows the Raman spectra after implementing polynomial background removal, which eliminates the background noise arising from the low optical density of the filters, using a second-order polynomial for this study.

### 3.2 Raman Spectra of Chemical Materials

**Fig. 3:**
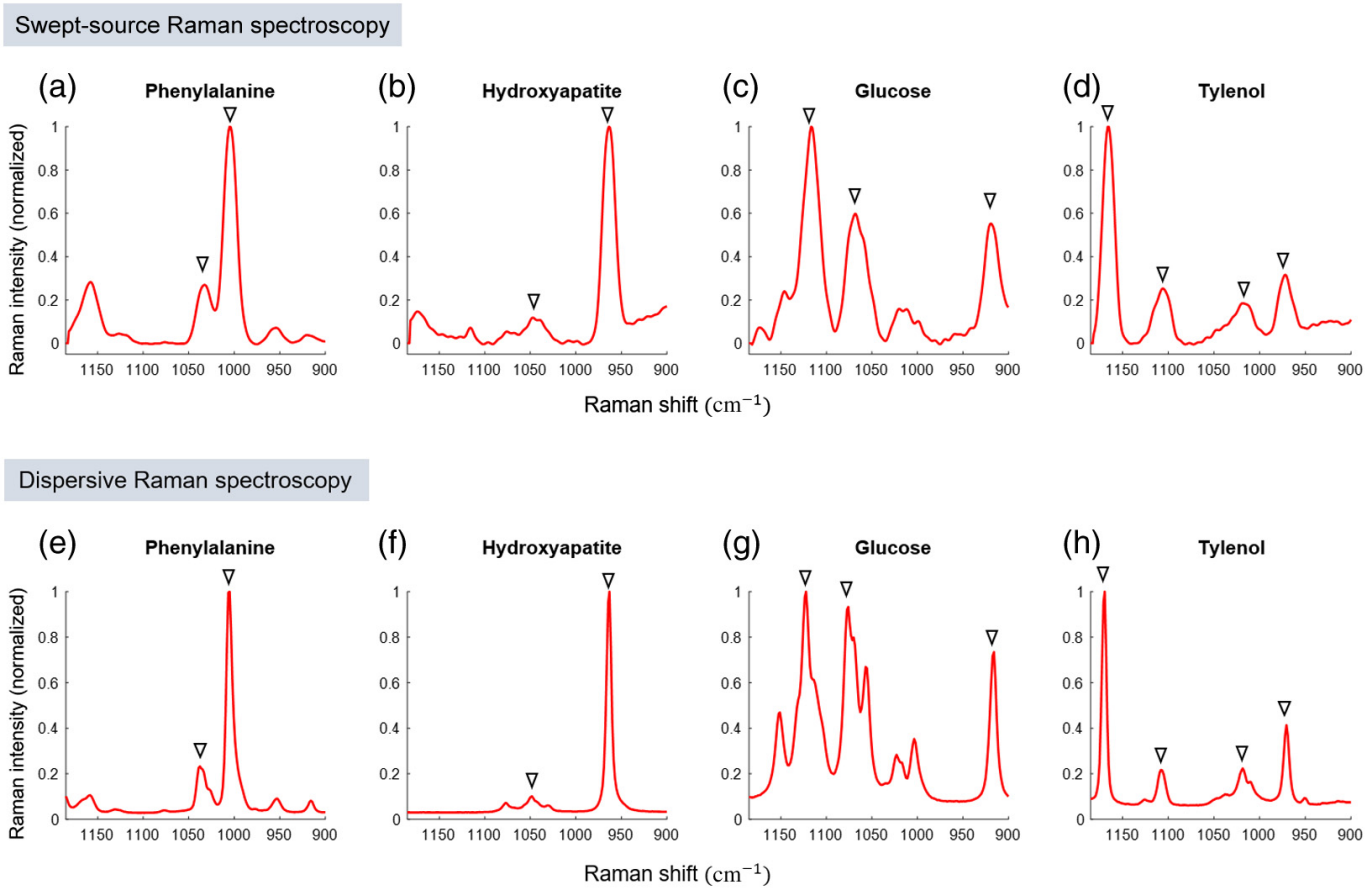
Raman spectra (∼900 to $1200{\;\mathrm{cm}}^{-1}$) of the chemical samples. (a)–(d) Raman spectra of phenylalanine, hydroxyapatite, glucose, and acetaminophen, respectively, obtained through the SS-Raman spectroscopy system, utilizing a wavelength sweep range from 822 to 842 nm, a power of 100 mW, and a wavelength sweep interval of 0.05 nm. (e)–(h) Corresponding Raman spectra obtained through dispersive Raman spectroscopy.

Raman spectroscopy measurements were conducted on chemical powder samples, including phenylalanine, hydroxyapatite, glucose, and acetaminophen, using a 100 mW power at the sample plane with postprocessing to enhance the spectral resolution. Figure 3 shows the Raman spectra of these chemical samples within the range of 900 to $1200\;{\mathrm{cm}}^{-1}$. Figures 3(a)–3(d) show the Raman spectra of the chemical samples obtained through the SS-Raman spectroscopy with notable Raman bands marked by triangle symbols. Figure 3(a) shows the Raman spectra of phenylalanine, with the Raman bands at 1030 and $1004\;{\mathrm{cm}}^{-1}$, assigned to the CH in-plane bending mode of the substituted benzene and the symmetric ring breathing mode, respectively.^25^^,^^26^ Figure 3(b) shows the Raman spectra of hydroxyapatite with Raman bands at 1045 and $960{\;\mathrm{cm}}^{-1}$, corresponding to the PO asymmetric valence and PO symmetrical valence mode, respectively.^27^ Figure 3(c) shows the Raman spectra of glucose with Raman bands at 1125, 1060, and $910\;{\mathrm{cm}}^{-1}$, associated with the COH bonds’ bending mode, CO stretching mode, and CH vibration, respectively.^28^^–^^30^ Figure 3(d) shows the Raman spectra of acetaminophen, featuring Raman bands at 1168 and $1104\;{\mathrm{cm}}^{-1}$, linked to the aromatic β CH bending. The Raman bands at 1016 and $968\;{\mathrm{cm}}^{-1}$ correspond to the ρ ${\mathrm{CH}}^{3}$ bending and aromatic γ CH bending, respectively.^31^ Figures 3(e)–3(h) show the Raman spectra of the same samples using dispersive Raman spectroscopy. We calculated the correlation coefficients between the Raman spectra obtained through the SS-Raman spectroscopy system and dispersive Raman spectroscopy for comparison. The correlation coefficients for phenylalanine, hydroxyapatite, glucose, and acetaminophen were 0.88, 0.84, 0.87, and 0.73, respectively, indicating a strong positive correlation.

### 3.3 Raman Spectra of Animal Tissue

The SS-Raman spectroscopy system was used to measure the Raman spectra of biological samples. The biological samples utilized in this study was purchased from a grocery store (Shaw’s Supermarket, Lynn, Massachusetts, United States). The samples were stored in a refrigerator at −18°C to prevent deformation. Because the researcher had no control over the premortem procedures or the process of euthanasia, occupational health risks were minimal; thus, the Institutional Animal Care and Use Committee at the Massachusetts Institute of Technology did not require a protocol.

**Fig. 4:**
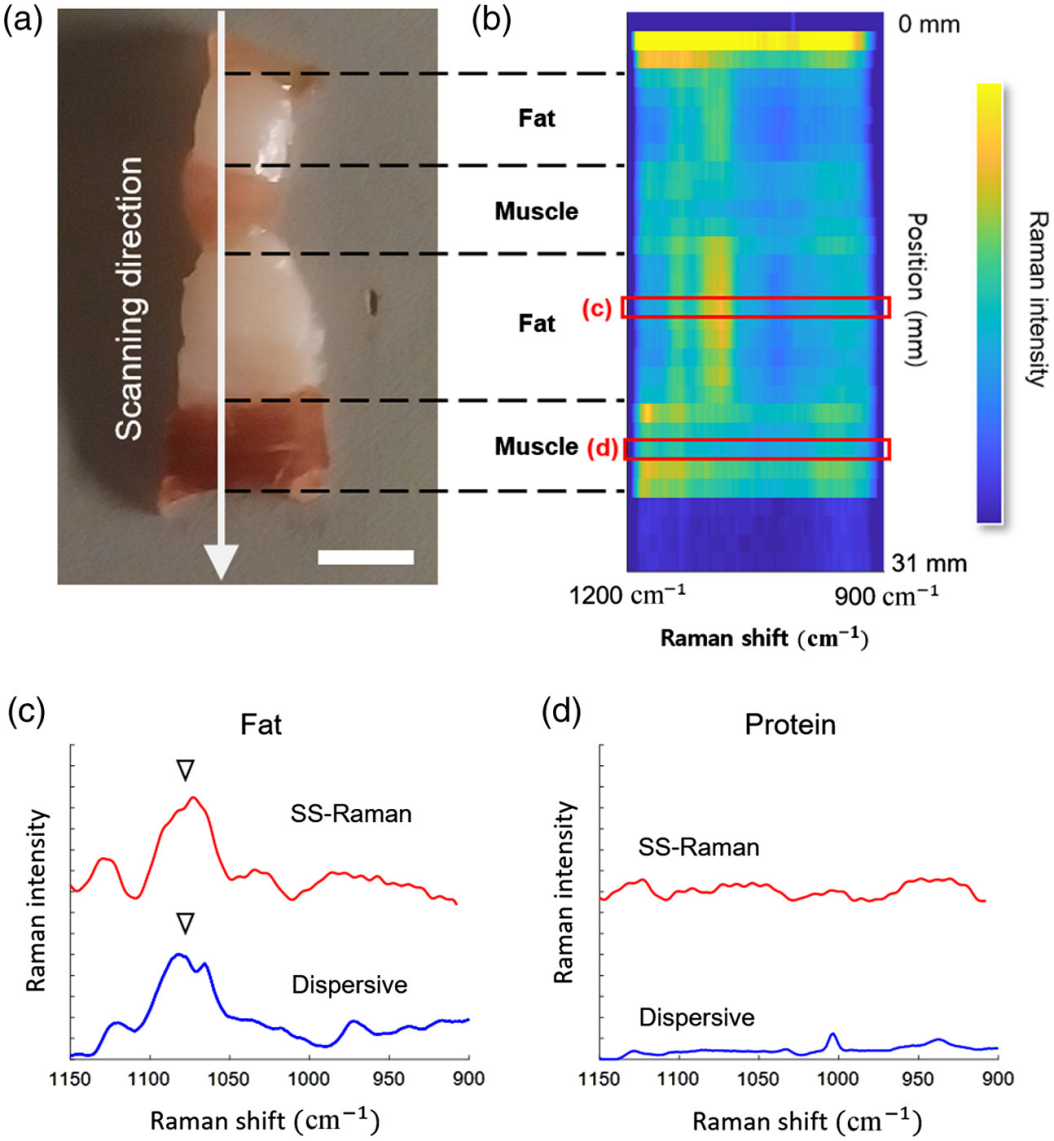
Raman spectra of biological samples obtained through SS-Raman spectroscopy. (a) Pork belly slice with the scanning direction indicated by the white arrow. The scale bar represents 5 mm. (b) The biological tissue mapping plot of sample composition. Raman spectra of (c) fat and (d) muscle layer, obtained through the SS-Raman spectroscopy (red) and dispersive Raman spectroscopy (blue).

In this study, pork belly slices with a thickness of 10 mm and a length of 25 mm were used as samples without any additional treatment. Raman spectra of the biological sample were acquired within the range of 900 to $1200\;{\mathrm{cm}}^{-1}$ using a 100 mW power at the sample plane. Each Raman spectrum was acquired with a 40 s acquisition time. Figure 4 presents the results of the biological tissue mapping plot using SS-Raman spectroscopy. The mapping was conducted on the cross-sections of the pork belly using a motorized stage. Figure 4(a) illustrates the scanning direction of the pork belly tissue, as indicated by a white arrow. The biological sample was scanned at 1 mm intervals over a length of 30 mm, resulting in the collection of 30 Raman spectra. Figure 4(b) shows the biological tissue mapping plot, providing information about the composition of the biological sample at each scanning location. Figure 4(c) shows the Raman spectra of the fat layer of the pork belly measured by SS-Raman and dispersive Raman spectroscopy, whereas Fig. 4(d) shows the Raman spectra of the muscle layer of the pork belly. The Raman spectra obtained through the SS-Raman spectroscopy are represented in red, and the Raman spectra obtained through dispersive Raman spectroscopy are depicted in blue. In general, fat has a larger Raman cross-section than that of protein. In the fat layer, a relatively intense Raman signal was observed at $1080\;{\mathrm{cm}}^{-1}$, assigned to the CC stretch mode of fat. Conversely, no clear Raman peak was observed in the muscle layer due to the low Raman cross-section and broadening of the sharp phenylalanine peak at $1004\;{\mathrm{cm}}^{-1}$. We calculated the correlation coefficient between the Raman spectra of the fat layer obtained through the SS-Raman and dispersive Raman spectroscopy for comparison. The correlation coefficient for the fat layer was 0.91, indicating a strong positive correlation.

## 4 Discussion and Conclusion

In this study, we demonstrated SS-Raman spectroscopy using a narrow bandwidth bandpass filter and a swept-source laser, creating a compact system without the need for a bulky spectrometer. Additionally, the use of a highly sensitive point detector enhances the sensitivity, representing an advantage of the proposed SS-Raman spectroscopy method. We validated the efficacy of our SS-Raman spectroscopy by acquiring Raman spectra from chemical specimens and providing Raman mapping of biological tissue.

However, despite the advancements in SS-Raman spectroscopy, currently, there are practical hardware limitations, particularly associated with the large bandwidth of the bandpass filter and the noise of the swept-source laser that compromise the spectral resolution. The large bandwidth filter in the SS-Raman spectroscopy enhances the throughput, which is especially advantageous for acquiring Raman signals from biological samples. However, there is a trade-off between throughput and spectral resolution. To address these limitations, we have applied Gaussian filtering to reduce high-frequency noise and performed deconvolution to enhance the spectral resolution. Moreover, we have incorporated polynomial background removal to remove background signals due to the low optical density of the filters, improving the spectral resolution by 8.5%, as measured by the FWHM calculations. The Raman spectra of the chemical samples shown in Fig. 3, including phenylalanine, hydroxyapatite, glucose, and acetaminophen, further corroborate our findings. Additionally, we validated our method with correlation coefficients of 0.88, 0.84, 0.87, and 0.73, respectively, when comparing the SS-Raman to dispersive Raman spectroscopy for these samples. We expect that the spectral resolution will be significantly improved by eliminating the broadening effects from other instruments.

Furthermore, we successfully conducted biological tissue mapping of pork belly slices using the SS-Raman spectroscopy with a motorized stage for point scanning across the tissues’ cross-sections. Through these Raman spectra, this technique enabled the clear differentiation between fat and muscle layers within the sample. Notably, the Raman spectra obtained using SS-Raman spectroscopy exhibited a high correlation with those obtained via dispersive Raman spectroscopy, achieving a correlation coefficient of 0.91, demonstrating the reliability and precision of the SS-Raman method in biological tissue characterization.

The SS-Raman spectroscopy developed in this feasibility study can be improved in spectral resolution, acquisition time, and collection efficiency. The large bandwidth of the bandpass filter (1.5 nm) currently limits the spectral resolution of the SS-Raman spectroscopy. However, recently released ultra-narrow bandpass filters with a bandwidth from 0.1 to 0.5 nm can enhance the spectral resolution of SS-Raman spectroscopy, achieving results comparable or even superior to the spectral resolution of the most dispersive Raman spectroscopy systems. The current acquisition time of 40 s for SS-Raman spectroscopy poses limitations in non-steady-state studies. To expedite the acquisition of high-quality Raman spectra, enhancing the measurement geometry of SS-Raman spectroscopy is imperative. Incorporating the confocal geometry with a high NA objective lens (0.95), as employed in the compared dispersive Raman spectroscopy, into the SS-Raman spectroscopy system can increase the collection efficiency by 14.5 times. Additionally, by mitigating background noise through the use of a high optical density (OD) optical filter and optimizing light source parameters such as the wavelength step interval and sweeping speed, the performance of SS-Raman spectroscopy can be significantly improved. Overall, we anticipate that the enhanced SS-Raman spectroscopy will enable the measurement of biological sample in <1  s. Moreover, the presented SS-Raman spectroscopy has a spectral range constrained by the laser’s sweeping capabilities. To overcome this, we are developing a multichannel SS-Raman system with multiple detectors and bandpass filters. Multichannel SS-Raman spectroscopy with four bandpass filters and detectors is expected to cover the entire fingerprint region within the same acquisition time. This enhancement will broaden the technique’s applicability across diverse application fields.

Recent trends in the field of Raman spectroscopy include the miniaturization of systems.^32^^–^^34^ We anticipate that SS-Raman spectroscopy could offer substantial advantages in miniaturization compared with dispersive methods. Furthermore, we are developing an OCT-Raman imaging system that simultaneously obtains morphological and chemical information. These efforts are consistent with advances in multimodal Raman spectroscopy, which integrates various imaging modalities. One example is a system that combines quantitative phase imaging and Raman functionality using a swept-source laser.^35^ We expect that the OCT-Raman system will be a pivotal tool in various biomedical applications, including cancer imaging and intravascular imaging.

## Data Availability

The datasets generated and/or analyzed during the current study are available from the corresponding author on reasonable request.
